# Sociodemographic Factors Influencing Knowledge, Attitudes, Practices, Glycaemic Control and Sight‐Threatening Retinopathy Among Diabetes Patients in Rawalpindi, Pakistan: A Hospital‐Based Cross‐Sectional Study

**DOI:** 10.1002/puh2.70384

**Published:** 2026-09-23

**Authors:** Raju Sapkota, M. Ahmed Abdullah, Hugo Onumajuru, Shahina Pardhan

**Affiliations:** ^1^ Vision & Eye Research Institute, School of Medicine, Faculty of Health, Medicine, and Social Care Anglia Ruskin University Cambridge UK; ^2^ Centre for Inclusive Community Eye Health, School of Medicine, Faculty of Health, Medicine, and Social Care, Anglia Ruskin University Cambridge UK; ^3^ Health Services Academy Islamabad Pakistan

**Keywords:** attitude, clinical outcomes, diabetes, knowledge, Pakistan, practice, sight‐threatening diabetic retinopathy

## Abstract

**Aim/objectives:**

To examine demographic factors influencing knowledge, attitude and practice (KAP), glycaemic control and sight‑threatening retinopathy (STR) among diabetes patients in Pakistan.

**Methods:**

A cross‐sectional study was conducted at Al‐Shifa Eye Trust Hospital, Rawalpindi, Pakistan, with 517 consecutive diabetes patients (mean age = 63.4 ± 10.5 years). A validated questionnaire in Urdu captured the KAP data. Data on fasting blood glucose (FBG), blood pressure (BP), and STR were also collected. Age, gender, literacy, employment status, diabetes duration, and treatment served as independent variables.

**Results:**

Participants showed limited understanding of the importance of controlling BP (51.3%) and cholesterol (64.1%), the risk of blindness due to diabetes (60.0%), and that a diabetic eye test differed from a routine eye test for spectacles (60.9%). These knowledge gaps were more common among older, female, and illiterate individuals. 70.7% of participants reported they would not seek an eye test if they were seeing well, with those with longer diabetes duration (>6 years) more likely to do so (OR = 1.05, *p* < 0.001). Older age and longer diabetes duration were significantly associated with reduced physical activity (*p* < 0.001). High FBG levels (>8.5 mmol/L) were significantly associated with older age, illiteracy, and longer diabetes duration. A total of 45.9% of participants had diabetic retinopathy, and 9.9% had STR. Older age (OR = 1.12, *p* < 0.001), longer diabetes duration (OR = 3.4, *p* = 0.04), and unemployment (OR = 6.2, *p* < 0.001) predicted STR significantly.

**Conclusion:**

Overall, participants showed inadequate KAP, uncontrolled diabetes, and a high diabetic retinopathy percentage. Older age and longer diabetes duration were consistently associated with poorer KAP, uncontrolled diabetes, and STR. The findings highlight the need for targeted diabetes education and screening programmes to improve awareness and early detection of diabetes‐related eye complications in Pakistan.

## Graphical abstract

Participants exhibited inadequate diabetes‐related knowledge, attitudes and practices (KAP), poor glycaemic control and a high prevalence of diabetic retinopathy. Older age and longer diabetes duration were consistently associated with poorer KAP, uncontrolled diabetes and sight‐threatening retinopathy.

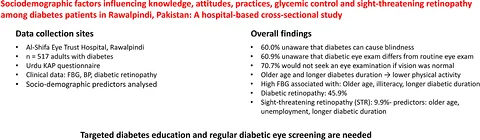

## 1 Introduction

Diabetes mellitus is a leading cause of morbidity and mortality worldwide, which is associated with serious health complications such as cardiovascular disease, retinopathy, nephropathy and neuropathy [1]. According to the International Diabetes Federation (IDF), approximately 589 million people were living with diabetes globally in 2024 [2]. Notably, more than three‐fourths of these individuals live in low‐ and middle‐income countries, with a significant concentration in South Asia, particularly in India and Pakistan [3, 4].

Pakistan ranks third globally in the prevalence of diabetes and second in South Asia after India, with over 34 million adult cases of diabetes in 2024 [2]. This figure is expected to rise to over 62 million by 2045 [2]. Furthermore, using secondary data and regression modelling techniques, Genitsaridi et al. [5] estimated that 11 million adults in Pakistan were living with impaired glucose tolerance in 2024, whereas a further 8.9 million were living with previously undiagnosed diabetes, that is, those who met the diagnostic criteria for diabetes on testing but had not previously been diagnosed. A systematic review and meta‐analysis conducted by Umar Hasan and Rehman Siddiqui [6] in 2024 reported the overall pooled prevalence of diabetes and prediabetes in Pakistan to be 10.0% and 11.0%, respectively. Notably, a higher prevalence of diabetes (32.9%) and prediabetes (37.4%) has been reported among the urban population [7]. Furthermore, the prevalence of diabetes is found to vary considerably across provinces, ranging from 13.2% in Khyber Pakhtunkhwa to 32.3% in Sindh, with prevalence estimates of 30.2% in Punjab and 29.5% in Balochistan [8]. The reasons for this substantial provincial variation remain unclear. However, differences in urbanization, socioeconomic status, lifestyle factors, obesity prevalence, and healthcare access are likely to contribute to these geographical disparities.

The high burden of diabetes in Pakistan and surrounding regions is driven by a combination of non‐modifiable factors, including age, family history of diabetes [9], and modifiable factors such as obesity [10], rapid urbanization [11], unhealthy dietary habits [12], lack of physical activity, particularly among women [13], limited access to healthcare facilities [14], and poor awareness about diabetes control [15]. Understanding the contribution of these interconnected factors is essential for identifying effective diabetes control interventions.

Although pharmacological intervention plays a central role in diabetes control, successful diabetes management also depends on improved knowledge, attitude, and practice (KAP) about the disease [16, 17]. Diabetes knowledge includes parameters such as an understanding of diabetes, its symptoms, complications, and risk factors such as family history, obesity, sedentary lifestyle, poor diet, age, ethnicity, the importance of monitoring blood glucose levels, and understanding recommended blood glucose targets. Diabetes attitudes include parameters such as perceived beliefs about diabetes and its treatment, willingness to adopt a healthy diet and exercise behaviour, and seeking social support from family members, healthcare providers and community members. Likewise, diabetes practice parameters include variables such as healthy eating (e.g., reducing sugar intake), doing regular physical exercise, adherence to a prescribed medication regimen, monitoring blood glucose levels regularly, attending diabetic eye screening appointments, reducing or quitting smoking and managing stress.

A hospital‐based study in Pakistan (*n* = 384) revealed that although nearly all patients were aware that blood glucose levels rise in diabetes, only half understood the risks associated with abnormal blood glucose levels [18]. Similarly, a population‐based survey (*n* = 139) found that only 18.7% of patients were aware of diabetes‐related complications such as eye, kidney and nerve problems [19]. Another hospital‐based study from Pakistan (*n* = 199) found that although men had better knowledge than women, only a small percentage (18.7%) of the overall participants had adequate knowledge about diabetic complications [20]. Misconceptions, such as believing that consuming bitter gourd could cure diabetes, were noted [20]. In another study (*n* = 230), only 50% of patients were able to correctly answer questions related to a healthy diet in diabetes, and just around 60% knew of the optimal blood glucose levels in diabetes treatment [21]. Younger patients, those with higher education levels and those who regularly attended diabetes clinics scored higher in the diabetes knowledge and awareness questionnaire overall [21].

Regarding diabetes self‐care practice, women in Pakistan were found to exhibit lower levels of physical activity, which has been attributed to cultural norms, household chores, and unsafe exercising environments [22, 23]. In addition, a hospital‐based study reported that only 58.5% of patients checked their blood glucose levels at least once every 3 months, whereas a population‐based study showed a much lower percentage of patients (8.6%) who regularly checked their blood glucose levels [24].

Although a large body of literature highlights inadequate diabetes‐related KAP among people in Pakistan, little is known about how different demographic factors influence KAP subcomponents and clinical findings such as fasting blood glucose (FBG), cholesterol levels and sight‐threatening diabetic retinopathy (STDR). Addressing this gap is essential for designing or refining person‐centred diabetes education programmes in Pakistan, which may be vital in reducing the burden of uncontrolled diabetes and STDR. The present study sought to address this gap.

## 2 Methodology

*Study design*: A hospital‐based cross‐sectional study. Data were collected between March and August 2023.

*Study site*: Al‐Shifa Eye Trust Hospital

*Study participants*: Five hundred and seventeen participants with Type 2 diabetes (mean age = 63.4 ± 10.5 years, range: 32–91 years) who presented consecutively at Al‐Shifa Eye Trust Hospital, Rawalpindi, Department of Endocrinology, Bahria International, were recruited. All participants gave informed consent to take part in the study. Participants were treated in accordance with applicable ethical guidelines that followed the tenets of the Helsinki Declaration. The study protocol was approved by the Institutional Review Board of Al‐Shifa Eye Trust Hospital (Ref. No. Fac‐IRB/03).

*Sample size calculation* **:**

$$n=\frac{z^{2}pq}{d^{2}};n=\frac{1.96^{2}\times 0.329\times \left(1-0.329\right)}{0.05^{2}}\approx 339$$

*n* = number of samples
*z* = 1.96 (95% confidence level)
*p *= prevalence estimate (32.9%, [7])
*q* = (1 − *p*)
*d* = Precession of the prevalence estimate

As we used the consecutive (non‐probability) sampling method, we collected data from significantly more samples (*n* = 517) compared to the minimum calculated sample size of 339 to maintain the study's strength. This sample size is larger than the sample size used in previous studies from Pakistan that explored diabetes‐related KAP [18, 19, 20, 21].

*Data collection*: A pre‐tested structured questionnaire interview, validated in previous studies [25, 26, 27], and a pro forma for recording clinical findings, including demographic information, duration of diabetes, type of treatment, and lifestyle factors was used. The questionnaire was translated into the Urdu language and then translated back into English, which was then reviewed by a dual‐language expert to ensure the accuracy of translation. All participants were able to respond to all the questions without any help. All the queries arising from the pretesting were addressed before the final data collection was conducted.

Interviews were conducted by a trained interviewer in comfortable environments to ensure that participants felt at ease and could express themselves freely. The interviewers ensured confidentiality and privacy during the interview process. Each interview session lasted approximately 30–45 min.

Blood tests were carried out on the day of the interview to measure key biomarkers, including FBG and the lipid profile, related to diabetes management. Trained healthcare professionals conducted the blood tests in a safe and hygienic manner to ensure accurate results under the supervision of the author (M.A.A.). Blood glucose levels above 8.5 mmol/L suggested uncontrolled glycaemic levels; the 8.5 mmol/L threshold was selected a priori on the basis of its use in previous studies [28]. Data on Glycated Haemoglobin (HbA1c) were not collected due to cost reasons.

Ophthalmological recordings were used to determine whether the patient had developed diabetic retinopathy and, for those with retinopathy, to classify it as sight‐threatening or non‐sight‐threatening based on the Early Treatment of Diabetic Retinopathy Studies (ETDRS) criteria [29]. Retinopathy severity of moderate non‐proliferative diabetic retinopathy or worse, and/or the presence of diabetic macular oedema characterized by retinal thickening within two‐disc diameters of the centre of the macula was categorized as sight‐threatening [30], otherwise, it was categorized as non‐sight‐threatening.

Data analysis: Data were analysed using SPSS (version 29). For convenience, participants’ raw data were binarized (e.g., yes, no/not sure; employed and unemployed). Age, gender, literacy status, employment status and diabetes duration were the independent variables, whereas KAP, FBG, blood pressure (BP), cholesterol and STDR were the dependent variables. Two participants had incomplete KAP data; therefore, data for some variables are based on 515 responses. Analyses were performed using listwise deletion method, with participants excluded from analyses requiring the missing variables. Data were assessed for normality and were analysed using parametric or non‐parametric tests, as appropriate. Associations between categorical variables such as gender, literacy level, employment status, treatment type, and duration of diabetes with parameters such as lifestyle practices (smoking, exercise and diet), knowledge/awareness and attitudes were examined using the chi‐square exact test. Independent‐sample *t*‐tests were used to compare continuous data such as age, number of episodes of uncontrolled diabetes, and the number of times patients forgot to take the diabetes medication in the previous month between binarized groups. Bonferroni correction was applied for multiple comparisons. Where two or more independent variables (demographics, duration and treatment of diabetes) were found to be statistically significant in the univariate analysis, they were used to perform multiple regression to determine which variables independently predicted the outcome measurements. Appropriate data reporting guidelines (Strengthening the Reporting of Observational Studies in Epidemiology, STROBE) were followed [31].

## 3 Results

### 3.1 Data on the Demographics, Duration and Type of Treatment

There were 277 males (53.6%, mean age = 62.9 years ±11.0) and 240 females (46.4%, mean age = 63.9 years ±9.9) participants. A total of 146 participants (28.2%) were not able to read or write English or Urdu (referred to as ‘illiterates’), and 312 (60.3%) were either employed or self‐employed. The self‐reported mean duration of diabetes of all participants was 6.7 years ±3.7, with 56.3% having diabetes for longer than 6 years. Out of the total participants, 40.8% reported being on insulin treatment (insulin only or insulin mixed with tablet treatment), and the remaining were on non‐insulin treatment (diet control only‐ 1.7% and tablet only‐ 57.5%). One‐third of the total participants (33.1%) stated that they were taking medication for high cholesterol and 43.7% for high BP. Table 1A provides a summary of demographics, diabetes duration, and type of treatment. Tables 1B and C provide a summary of the KAP and clinical parameters.

**TABLE 1:** Overall summary of independent (A) and the dependent variables related to diabetes knowledge, attitude and practice (KAP) (B) and clinical parameters (C).

Age (mean ± SD)	63.4 years ± 10.5		
Variable	Category	Number	%
**A. Independent variables**			
Gender	Male	277	53.6
	Female	240	46.4
Ability to read and write English or Urdu	Yes (literate)	369	71.7
	No (illiterate)	146	28.3
Employment status	Employed	225	43.5
	Unemployed	205	39.7
	Self‐employed	87	16.8
Type of diabetes treatment	Diet only	9	1.7
	Tablet only	296	57.5
	Insulin only	142	27.6
	Tablet/insulin mixed	68	13.2
Duration of diabetes	Up to 6 years	225	43.7
	>6 years	290	56.3
Use of medication for high blood pressure	Yes	226	43.7
	No	291	56.3
Use of medication for high cholesterol	Yes	171	33.1
	No	346	66.9
**B. KAP question**			
*Knowledge*			
Is BP control important for managing diabetes?	Yes	251	48.7
	No	147	28.6
	I don't know	117	22.7
Is cholesterol control important for managing diabetes?	Yes	185	35.9
	No	171	33.2
	I don't know	159	30.9
Is diabetes a serious disease?	Yes	348	67.6
	No	117	22.7
	I don't know	50	9.7
Is exercise important to control your diabetes?	Yes	289	56.1
	No	176	34.2
	I don't know	50	9.7
Is smoking harmful to your diabetes?	Yes	337	65.4
	No	143	27.8
	I don't know	35	6.8
Can diabetes cause blindness?	Yes	197	38.3
	No	152	29.5
	I don't know	166	32.2
Is there a difference between having your eyes tested for spectacles and diabetes?	Yes	201	39.0
	No	232	45.1
	I don't know	82	15.9
Is the control of diabetes your responsibility or your doctor's, or the responsibility of both?	Doctor's	177	34.4
	Mine	209	40.6
	Both mine and the doctor's doctor	99	19.2
	I don't know	30	5.8
*Attitude*			
Would you be comfortable telling people that you have diabetes (do not count doctors, nurses)?	Yes	347	67.4
	No	163	31.6
	I don't know	5	1.0
Is it difficult to maintain a healthy diet during weddings and festivals?	Yes	336	65.2
	No	168	32.6
	I don't know	11	2.1
Would you go to an eye doctor to have your eyes tested if you were seeing well?	Yes	151	29.3
	No	334	64.9
	I don't know	30	5.8
*Practice*			
Do you exercise?	Yes	112	21.7
	No	395	76.7
	I don't know	8	1.6
Do you smoke?	Yes	184	35.7
	No	331	64.3
When did you last have your eyes checked to see if diabetes affected your eyes?	Within a month	21	4.1
	Within 6 months	117	22.7
	Within 1 year	180	35.0
	Within 2 years	72	14.0
	Never	125	24.3
How often last year did you go to a hospital or doctor because your blood sugar was uncontrolled?	None	4	0.8
	1–10 times	257	49.9
	10–20 times	218	42.3
	≥20 times	36	7.0
**C. Clinical variable**	**Mean ± SD**		
FBG	8.4 ± 1.3 mmol/L		
HDL	59.07 ± 11.2 mg/dL		
LDL	84.6 ± 23.8 mg/dL		
Systolic BP	124.5 ± 17.1 mmHg		
Diastolic BP	78.6 ± 12.3 mmHg		
**Retinopathy status**	**Number of patients (%)**		
Sight‐threatening	51 (9.9)		
Non‐sight threatening	186 (36.0)		
Normal (no retinopathy present)	265 (51.2)		
Could not be determined	15 (2.9)		

Abbreviations: BP, blood pressure; FBG, fasting blood glucose.

### 3.2 Overall KAP Data

Out of the total participants, more than half reported not knowing that BP (51.3%) and cholesterol control (64.1%) were important in diabetes management. Nearly one‐third (32.4%) were not aware that diabetes is a serious disease. As high as 61.7% were not aware that uncontrolled diabetes can cause blindness. Nearly a quarter (24.3%) reported never attending a diabetic eye test. Only 19.2% reported that diabetes management required self‐management as well as care offered by their doctor. A significant percentage (43.9%) were not aware that physical activity was important in diabetes control. Over one‐third (35.7%) said they smoked, and 34.6% were unaware that smoking is harmful to diabetes control.

### 3.3 Overall Clinical Data

Participants presented with mean FBG levels of 8.4 ± 1.3 mmol/L, which is higher than the criterion level of ≥7 mmol/L for diabetes [28]. Diabetic retinopathy was identified in 45.9% of participants, of which 9.9% had a sight‐threatening form. In a small minority (2.9%), retinopathy grading was not possible, mostly due to dense cataracts.

Next, the associations between each independent variable (age, gender, literacy status, employment status, duration of diabetes, and the type of treatment) and the dependent variable of diabetes‐related knowledge were examined. Results are provided in Table 2.

**TABLE 2:** Association of demographic variables, including the duration and the type of treatment, with diabetes‐related knowledge.

			Gender		Literacy level		Employment status		Type of treatment		Duration of diabetes	
Questions	Age (years) mean ± SD	Categories	Male	Female	Literate	Illiterate	Employed	Unemployed	Insulin/mix	Non‐insulin	Up to 6 years	>6 years
Is BP control important for managing diabetes?	61.3 ± 10.9	Yes	46.2%	51.7%	55.6%	31.5%	50.3%	46.3%	36.2%	57.4%	55.6%	43.4%
	65.1 ± 9.6	No/Not sure	53.8%	48.3%	44.4%	68.5%	49.7%	53.7%	63.8%	42.6%	44.4%	56.6%
	*p* value <0.001^*^	*p* value	0.22		<0.001^*^		0.42		<0.001^*^		0.008^*^	
Is cholesterol control important for managing diabetes?	59.5 ± 10.5	Yes	34.2%	37.9%	39.3%	27.4%	42.0%	26.2%	31.0%	39.3%	43.6%	30.0%
	65.4 ± 9.8	No/Not sure	65.8%	62.1%	60.7%	72.6%	58.0%	73.4%	69.0%	60.7%	56.4%	70.0%
	*p* value <0.001^*^	*p* value	0.41		0.01^*^		<0.001^*^		0.06		0.002^*^	
Is diabetes a serious disease?	62.1 ± 10.3	Yes	62.5%	73.3%	70.5%	60.3%	66.7%	69.0%	61.4%	71.8%	71.1%	64.8%
	65.8 ± 10.2	No/Not sure	37.5%	26.7%	29.5%	39.7%	33.3%	31.0%	38.6%	28.2%	28.9%	35.2%
	*p* value <0.001^*^	*p* value	0.01^*^		0.03^*^		0.63		0.02^*^		0.16	
Is the control of diabetes yours, the doctor's or the responsibility of both?	64.6 ± 10.0	Doctor's/mine/don't know	74.5%	87.9%	75.9%	93.2%	78.5%	84.2%	87.1%	76.4%	74.7%	85.5%
	57.6 ± 10.2	Both mine and the doctor's	25.5%	12.1%	24.1%	6.8%	21.5%	15.8%	12.9%	23.6%	25.3%	14.5%
	*p* value <0.001^*^	*p* value	<0.001^*^		<0.001^*^		0.11		0.003^*^		0.002^*^	
Is exercise important to control your diabetes?	57.4 ± 10.0	Yes	25.5%	17.5%	24.9%	13.7%	25.6%	15.8%	11.4%	28.9%	31.1%	14.5%
	64.9 ± 10.0	No/Not sure	74.5%	82.5%	75.1%	86.3%	74.4%	84.2%	88.6%	71.1%	68.9%	85.5%
	*p* value <0.001^*^	*p* value	0.03^*^		0.006^*^		0.009^*^		<0.001^*^		<0.001^*^	
Is smoking harmful to your diabetes?	62.2 ± 10.5	Yes	72.0%	57.9%	65.6%	65.1%	63.5%	36.5%	49.5%	76.4%	63.6%	66.9%
	65.3 ± 10.1	No/Not sure	28.0%	42.1%	34.4%	34.9%	36.5%	31.5%	50.5%	23.6%	36.4%	33.1%
	*p* value = 0.001^*^	*p* value	<0.001^*^		0.92		0.26		<0.001		0.46	
Can diabetes cause blindness?	61.2 ± 11.4	Yes	44.4%	31.3%	44.4%	22.6%	35.3%	42.5%	30.5%	43.6%	37.3%	39.0%
	64.6 ± 9.6	No/Not sure	55.6%	68.8%	55.6%	77.4%	64.7%	57.1%	69.5%	56.4%	62.7%	61.0%
	*p* value <0.001^*^	*p* value	0.003^*^		<0.001^*^		0.10		0.003^*^		0.72	
Is there a difference between eye tests for spectacles and diabetes?	61.6 ± 11.3	Yes	38.2%	40.0%	48.5%	15.1%	45.8%	28.6%	36.2%	41.0%	41.8%	36.9%
	64.4 ± 9.7	No/Not sure	61.8%	60.0%	51.5%	84.9%	54.2%	71.4%	63.8%	59.0%	58.2%	63.1%
	*p* value = 0.003^*^	*p* value	0.72		<0.001^*^		<0.001^*^		0.31		0.28	

*Note:* Columns add to 100% for gender, literacy level, employment status, type of treatment and duration of diabetes.

Abbreviation: BP, blood pressure.

* Represents a significant *p* value.

Variables with statistically significant associations were included in the multiple regression analysis. Detailed results are provided in Supporting Information File S1. Literate individuals were significantly more likely to know correct answers to diabetes‐related knowledge components, for example, (i) BP (OR = 2.5, *p* < 0.001, 95% CI: 1.6–3.7) and cholesterol (OR = 1.5, *p* = 0.05, 95% CI: 1.3–1.8) control are important in diabetes management; (ii) diabetes is a serious disease (OR = 1.5, *p* = 0.04, 95% CI: 1.02–2.35); (iii) uncontrolled diabetes may cause blindness (OR = 2.4, *p* < 0.001, 95% CI: 1.56–3.83) and (iv) the control of diabetes is a shared responsibility of the patient and the doctor (OR = 3.7, *p* < 0.001, 95% CI: 1.81–7.45). In addition, males were significantly more likely to know that diabetes can cause blindness (OR = 1.6, *p* = 0.01, 95% CI: 1.10–2.32) and that smoking is harmful in diabetes (OR = 1.8, *p* = 0.004, 95% CI: 1.22–2.61). Employed participants were significantly more likely to know that good cholesterol control was important in diabetes management (OR = 1.5, *p* = 0.05, 95% CI: 1.0–2.5). Likewise, employed participants were significantly more likely to know that a diabetic eye test is different from a routine eye test for spectacles (OR = 1.8, *p* = 0.004, 95% CI: 1.2–2.7). In addition, those on non‐insulin treatment were significantly more likely to know that BP control is important in diabetes management (OR = 1.8, *p* = 0.004, 95% CI: 1.2–2.6), that smoking is harmful to diabetes (OR = 2.9, *p* < 0.001, 95% CI: 2.0–4.46) and that exercise is important in controlling diabetes (OR = 1.8, *p* = 0.04, 95% CI: 1.07–3.1).

Table 3 shows how independent variables, such as age, gender, literacy status, employment status, duration of diabetes, and the type of treatment, were associated with the dependent variable of diabetes‐related attitudes.

**TABLE 3:** Association of demographic variables, including the duration and the type of treatment, with diabetes‐related attitude.

			Gender		literacy level		Employment status		Type of treatment		Duration of diabetes	
Questions	Age (years) mean ± SD	Categories	Male	Female	Literate	Illiterate	Employed	Unemployed	Insulin/mix	Non‐insulin	Up to years	>6 years
Would you be comfortable telling people that you have diabetes (do not count doctors or nurses)?	63.4 ± 10.3	Yes	66.9%	67.9%	68.6%	64.4%	67.3%	67.5%	67.1%	67.5%	66.2%	68.3%
	63.1 ± 10.8	No/Not sure	33.1%	32.1%	31.4%	35.6%	32.7%	32.5%	32.9%	32.5%	33.8%	31.7%
	*p* value = 0.8	*p* value	0.85		0.4		0.99		0.92		0.64	
Would you go to have your eyes tested if you were seeing well?	62.1 ± 10.8	Yes	28.0%	30.8%	32.0%	22.6%	34.7%	25.2%	25.2%	32.1%	36.9%	23.4%
	64.1 ± 10.2	No/Not sure	72.0%	69.2%	68.0%	77.4%	65.3%	74.8%	74.8%	67.9%	63.1%	76.6%
	*p* value = 004^*^	*p* value	0.5		0.04^*^		0.02^*^		0.10		0.001^*^	
Is it difficult to maintain a healthy diet at weddings and festivals?	62.2 ± 10.9	Yes	65.5%	65.0%	67.5%	59.6%	69.8%	61.7%	60.5%	68.5%	68.4%	62.8%
	65.2 ± 9.2	No/Not sure	34.5%	35.0%	32.5%	40.4%	30.2%	38.2%	39.5%	31.5%	31.6%	37.2%
	*p* value = 0.002^*^	*p* value	0.93		0.1		0.06		0.06		0.19	

*Note:* Columns add to 100% for gender, literacy level, employment status, type of treatment, and duration of diabetes.

* Represents a significant *p* value.

Variables with statistically significant associations were included in the multiple regression analysis. Results showed that those with a diabetes duration of up to 6 years were significantly more likely to go for an eye test, even if they saw well (OR = 1.05, *p* < 0.001, 95% CI: 1.03–1.08).

Table 4 shows how independent variables, such as age, gender, literacy status, employment status, duration of diabetes, and type of treatment, were associated with the dependent variable of diabetes‐related self‐care practices.

**TABLE 4:** Association of demographic variables, including the duration and the type of treatment with diabetes‐related practice.

			Gender		literacy level		Employment status		Type of treatment		Duration of diabetes	
Questions	Age (years) mean ± SD	Categories	Male	Female	Literate	Illiterate	Employed	Unemployed	Insulin/Mix	Non‐insulin	Up to 6 years	>6 years
How often last year did you go to a hospital or doctor because your blood sugar was uncontrolled?	60.8 ± 10.4	None	51.6%	49.6%	56.4%	36.3%	62.8%	32.0%	41.4%	57.0%	64.9%	39.7%
	65.9 ± 9.9	At least once	48.4%	50.4%	43.6%	63.7%	37.2%	68.0%	58.6%	43.0%	35.1%	60.3%
	*p* value <0.001^*^	*p* value	0.66		<0.001^*^		<0.001^*^		<0.001^*^		<0.001^*^	
Have you told your family members that you have diabetes?	63.3 ± 10.8	Yes	79.6%	74.6%	77.0%	78.1%	75.0%	80.8%	73.3%	80.0%	75.1%	79.0%
63.1 ± 9.1	No	20.4%	25.4%	23.0%	21.9%	25.0%	19.2%	26.7%	20.0%	24.9%	21.0%
	*p* value = 0.84	*p* value	0.21		0.82		0.13		0.09		0.34	
Do you exercise?	57.4 ± 10.0	Yes	25.5%	17.5%	24.9%	13.7%	25.6%	15.8%	11.4%	28.9%	31.1%	14.5%
	64.9 ± 10.0	No	74.5%	82.5%	75.1%	86.3%	74.4%	84.2%	88.6%	71.1%	68.9%	85.5%
	*p* value <0.001^*^	*p* value	0.03^*^		0.006^*^		0.009^*^		<0.001^*^		<0.001^*^	
Do you smoke?	59.7 ± 9.5	Yes	44.0%	26.3%	38.5%	28.8%	43.9%	23.2%	28.6%	40.7%	47.1%	26.9%
	65.3 ± 10.4	No	56.0%	73.8%	61.5%	71.2%	56.1%	76.8%	71.4%	59.3%	52.9%	73.1%
	*p* value <0.001^*^	*p* value	<0.001^*^		0.04^*^		0.001^*^		0.005^*^		<0.001^*^	
When did you last have an eye check‐up to see if diabetes affected your eyes?	60.4 ± 10.6	Within the last year	36.7%	37.9%	36.6%	39.0%	38.5%	35.5%	29.5%	42.6%	47.1%	29.7%
	65.0 ± 9.9	less frequently or never	63.3%	62.1%	63.4%	61.0%	61.5%	64.5%	70.5%	57.4%	52.9%	70.3%
	*p* value <0.001^*^	*p* value	0.79		0.61		0.52		0.003^*^		<0.001^*^	

*Note:* Columns add to 100% for gender, literacy level, employment status, type of treatment, and duration of diabetes.

* Represent a significant *p* value.

Variables with statistically significant associations were included in the multiple regression analysis. Detailed results are provided in Supporting Information File S2. Results showed that younger participants were significantly more likely to exercise (OR = 1.06, *p* < 0.001, 95% CI: 1.04–1.09), smoke (OR = 1.04, 95%, *p* < 0.001, 95% CI: 1.02–1.06) and go for diabetic eye check‐ups annually (OR = 1.03, 95% CI: 1.01–1.06). Older participants (OR = 1.03, *p* = 0.01, 95% CI: 1.0–1.05), illiterates (OR = 2.1, *p* < 0.001, 95% CI: 1.4–3.3), unemployed individuals (OR = 1.7, *p* = 0.007, 95% CI: 1.16–2.56) and those with diabetes duration longer than 6 years (OR = 2.2, *p* < 0.001, 95% CI: 1.47–3.32) were significantly more likely to have episodes of uncontrolled blood sugar levels for which they had to visit the hospital or a doctor.

Table 5 shows how independent variables, such as age, gender, literacy status, employment status, duration of diabetes, and type of treatment, were associated with diabetes‐related clinical parameters such as FBG, BP, cholesterol, and retinopathy severity.

**TABLE 5:** Association of independent variables with clinical findings.

	Gender		literacy level		Employment status		Type of treatment		Duration of diabetes	
Clinical measurements (mean ± SD)	Male	Female	Literate	Illiterate	Employed	Unemployed	Insulin/Mix	Non‐insulin	Up to years	>6 years
FBG (mmol/L)	8.5 ± 1.3	8.4 ± 1.2	8.4 ± 1.2	8.7 ± 1.4	8.2 ± 1.0	8.7 ± 1.5	8.7 ± 1.6	8.2 ± 0.9	8.2 ± 1.0	8.7 ± 1.4
*p* value	0.76		0.01^*^		<0.001^*^		<0.001^*^		<0.001^*^	
HDL (mg/dL)	58.7 ± 10.7	59.5 ± 11.8	58.5 ± 11.3	60.5 ± 10.9	59.8 ± 11.7	57.9 ± 10.4	60.8 ± 10.8	57.8 ± 11.4	57.7 ± 10.9	60.1 ± 11.4
*p* value	0.4		0.08		0.07		0.003^*^		0.02^*^	
LDL (mg/dL)	86.3 ± 23.7	82.6 ± 23.8	85.9 ± 22.9	81.1 ± 25.7	83.5 ± 23.9	86.1 ± 24.7	78.5 ± 25.1	88.7 ± 22.0	89.0 ± 24.9	81.0 ± 22.4
*p* value	0.08		0.04^*^		0.22		<0.001^*^		<0.001^*^	
Systolic BP (mmHg)	124.5 ± 16.7	124.6 ± 17.6	125.7 ± 17.7	121.4 ± 15.3	121.9 ± 15.1	128.5 ± 19.2	119.8 ± 17.4	127.7 ± 16.2	124.2 ± 15.8	124.6 ± 18.1
*p* value	0.94		0.01^*^		<0.001		<0.001^*^		0.77	
Diastolic BP (mmHg)	79.0 ± 11.7	78.5 ± 12.0	79.1 ± 12.1	77.9 ± 11.2	77.8 ± 11.7	80.3 ± 11.9	77.1 ± 13.1	80.0 ± 10.8	79.5 ± 12.0	78.0 ± 12.6
*p* value	0.66		0.3		0.009^*^		0.01^*^		0.81	
BMI	28.2 ± 13.2	28.4 ± 10.7	28.4 ± 13.9	28.0 ± 5.5	28.6 ± 15.1	27.7 ± 4.7	28.2 ± 5.7	28.4 ± 15.0	29.0 ± 17.2	27.8 ± 5.4
*p* value	0.86		0.75		0.42		0.91		0.27	
STDR (*n* = 51)	23.7%	18.4%	18.3%	26.8%	5.0%	38.8%	33.6%	7.1%	4.4%	31.0%
NSTDR (*n* = 186)	76.3%	81.6%	81.7%	73.2%	95.0%	61.2%	66.4%	92.9%	95.6%	69.0%
*p* value	0.34		0.16		<0.001^*^		<0.001^*^		<0.001^*^	

Abbreviations: BP, blood pressure; FBG, fasting blood glucose; STDR, sight‐threatening diabetic retinopathy.

* Represents a significant *p* value.

FBG, HDL and LDL were found to be positively correlated with older age (*p* < 0.05). A statistically significant difference in the mean age of those with and without STDR was also found (*p* < 0.05). Multiple linear regression showed a statistically significant relationship of higher FBG with age greater than 60 years (*t* = 2.5, *p* = 0.01), longer duration of diabetes (*t* = 3.1, *p* = 0.002), being unemployed (*t* = 3.0, *p* = 0.003), and the use of insulin (*t* = 2.6, *p* = 0.01). A multiple logistic regression showed older age (OR = 1.1, 95% CI: 0.85–0.93, *p* < 0.001), duration of diabetes longer than 6 years (OR = 3.4, 95% CI: 1.03–11.04, *p* = 0.04) and unemployment (OR = 6.2, 95% CI: 2.24–17.03, *p* < 0.001) to predict STDR independently (Nagelkerke *R* Square = 0.51).

## 4 Discussion

Given the limited evidence available from Pakistan, this study examined how demographic factors, as well as the duration of diabetes and the type of treatment, independently influence subcomponents of diabetes‐related KAP among hospital‐attending patients in Rawalpindi, Pakistan. In addition, the study also explored how these factors influenced clinical parameters such as blood glucose levels, BP, cholesterol, and STDR.

Our findings highlight significant gaps in diabetes‐related KAP among participants. The findings support prior reports of lack of knowledge and awareness about diabetes in Pakistan [20, 24, 30] and also provide a granular analysis of which diabetes‐related KAP subcomponents are linked independently with participants’ demographics, duration of diabetes and type of diabetic treatment. Knowledge/awareness data suggested that interventional programmes aimed at improving understanding about diabetes should target specific demographics (age, gender, literacy level and employment status), duration of diabetes and type of treatment. For illiterate and older individuals, this may include emphasizing the seriousness of the disease, the importance of controlling BP and cholesterol levels, and knowing that diabetes control requires joint efforts from the patient and the doctor. The targeted need to improve awareness of the importance of regular diabetic eye check‐ups among illiterate individuals was also noted. A previous study by Shera et al. [9] highlighted a significant lack of diabetes knowledge among individuals with lower literacy levels, particularly in rural areas of Pakistan.

Furthermore, our findings revealed that gender‐specific educational interventions are crucial, as female participants were less aware of the importance of physical exercise, that control of diabetes is a shared responsibility of them and their doctor, that smoking is harmful to diabetes, and that diabetes can cause blindness. Previous studies have reported that women in Pakistan are less likely to engage in physical exercise due to cultural and household responsibilities [21, 22].

Our findings also highlight the need to educate those with longer (>6 years) diabetes duration about the importance of controlling BP and cholesterol levels. Furthermore, elderly participants and those with a diabetes duration longer than 6 years were more likely not to go for diabetic eye check‐ups, even if they were seeing well, despite being at increased risk of developing STDR [32]. Beyond diabetes duration, several demographic and lifestyle factors were associated with poorer self‐care practices. Although smoking was more prevalent among younger individuals, in males and those with a shorter duration of diabetes, lower levels of physical activity were observed among older individuals and those receiving insulin treatment. These findings highlight the importance of promoting physical activity among people with diabetes, particularly older adults and individuals on insulin treatment. Smoking cessation interventions should be prioritized for younger patients, in men, and those with a shorter duration of diabetes.

Our findings of a mean FBG level of 8.4 mmol/L indicate suboptimal glycaemic control when compared to the accepted optimal level of <7 mmol/L [33], with 37.8% of participants having FBG levels indicative of uncontrolled diabetes (>8.5 mmol/L) [28]. Similar findings have been reported in Pakistan, where only 17% of patients achieved optimal glycaemic control, particularly among those with lower educational attainment [34]. In our study, illiteracy, unemployment, longer diabetes duration (>6 years) and insulin treatment were associated with poorer glycaemic control. The poorer control observed among insulin users may reflect both advanced disease and challenges related to insulin affordability and availability in Pakistan [35]. Consistent with previous research [20], longer diabetes duration was also associated with higher FBG levels.

Our study has some limitations. Reliance on self‐reports may introduce potential bias. Additionally, the cross‐sectional design restricted the ability to infer causality, highlighting the need for longitudinal studies to confirm these findings and explore the long‐term influence of demographic factors on diabetes‐related KAP and clinical outcomes. Moreover, HbA1c levels were not measured, which is a preferred method of diagnosing and monitoring diabetes. Although the sample size was robust, it was limited to a specific population in Rawalpindi, which may affect the generalizability of the results to the broader population in Pakistan.

In conclusion, this study found that various sociodemographic and clinical factors, including diabetes duration and treatment type, were independently associated with diabetes‐related KAP and clinical outcomes. Individuals who were illiterate, unemployed or had diabetes for longer than 6 years generally exhibited poorer knowledge and suboptimal diabetes control. Older participants were less likely to undergo annual diabetic eye screening and engaged in lower levels of physical activity, whereas younger participants were more likely to smoke. These findings suggest that age influences different aspects of diabetes self‐management and support the need for age‐specific behavioural interventions. Furthermore, our findings underscore the importance of targeted, patient‐centred diabetes education strategies that address the specific needs of high‐risk groups. Furthermore, guided by the Health Belief Model [36], our results suggest that improving awareness of the consequences of diabetic complications, particularly STDR, and emphasizing the benefits of healthy lifestyle behaviours may promote diabetes self‐management, improve clinical outcomes, and reduce the risk of blindness in Pakistan.

## Author Contributions

Raju Sapkota, Shahina Pardhan and M. Ahmed Abdullah contributed to the study's conception and design. M. Ahmed Abdullah applied and obtained ethics approval for the study. Data collection was performed by M. Ahmed Abdullah. Data analysis was performed by Raju Sapkota and Hugo Onumajuru. The first draft of the manuscript was written by Raju Sapkota, and all authors commented on previous versions of the manuscript. All the authors have read and approved the final manuscript.

## Funding

The authors have nothing to report.

## Ethics Statement

All methods were carried out following relevant guidelines and regulations that followed the tenets of the Helsinki Declaration. The study protocol was approved by the Institutional Review Board of Al‐Shifa Eye Trust Hospital (Ref. No. Fac‐IRB/03).

## Consent

All participants gave informed consent to take part in the study.

## Conflicts of Interest

The authors declare no conflicts of interest.

## Supporting information




**Supporting File 1**: puh270384‐sup‐0001‐SuppMat.docx

**Supporting File 2**: puh270384‐sup‐0002‐SuppMat.docx

## Data Availability

Data are available on request from the authors.
