# Supplementary material for: Sociodemographic Factors Influencing Knowledge, Attitudes, Practices, Glycaemic Control and Sight‐Threatening Retinopathy Among Diabetes Patients in Rawalpindi, Pakistan: A Hospital‐Based Cross‐Sectional Study
Source: Public Health Chall. 2026 Sep 23;5(3):e70384. doi: 10.1002/puh2.70384 (PMC13600724; doi:10.1002/puh2.70384)
Supplement: Supplementary file 1 [file PUH2-5-e70384-s001.docx]

**Supplementary file 1.** Results of multiple regression analysis showing significant predictor variables of diabetes-related knowledge. * represents a significant p-value.

| ***(B). Multiple regression analysis:*** | | | |  |  |  |  |  |
| --- | --- | --- | --- | --- | --- | --- | --- | --- |
| **Dependent/outcome variable** | **Predictor variable** | **Odds ratio (95% CI)** | **P-value** |  |  |  |  |  |
| Knowing that BP control is important in diabetes management | Younger age | 1.03 (1.0-1.05) | 0.02* |  |  |  |  |  |
|  | literate | 2.5 (1.6-3.7) | <0.001* |  |  |  |  |  |
|  | Non-insulin treatment | 1.8 (1.2-2.6) | 0.004* |  |  |  |  |  |
| Knowing that cholesterol control is important in diabetes management | Younger age | 1.05 (1.03-1.08) | <0.001* |  |  |  |  |  |
|  | literate | 1.5 (1.3-1.8) | 0.05* |  |  |  |  |  |
|  | Being employed | 1.5 (1.0-2.5) | 0.05* |  |  |  |  |  |
| Knowing that diabetes is a serious disease | Younger age | 1.03 (1.01-1.05) | 0.002* |  |  |  |  |  |
|  | literate | 1.5 (1.02-2.35) | 0.04* |  |  |  |  |  |
|  | Female | 1.8 (1.25-2.71) | 0.002* |  |  |  |  |  |
| Knowing that the control of diabetes is the responsibility of both the doctor and the patient | Younger age | 1.06 (1.04-1.09) | <0.001* |  |  |  |  |  |
|  | literate | 3.7 (1.81-7.45) | <0.001* |  |  |  |  |  |
|  | Male | 2.2 (1.34-3.65) | 0.02* |  |  |  |  |  |
| Knowing that exercise is important to control diabetes | Younger age | 1.06 (1.04-1.09) | <0.001* |  |  |  |  |  |
|  | Non-insulin treatment | 1.8 (1.07-3.1) | 0.04* |  |  |  |  |  |
| Knowing that smoking is harmful to diabetes | Male | 1.8 (1.22-2.61) | 0.004* |  |  |  |  |  |
|  | Non-insulin treatment | 2.9 (2.0-4.46) | < 0.001* |  |  |  |  |  |
| Knowing that diabetes can cause blindness | Younger age | 1.03 (1.0-1.05) | 0.01* |  |  |  |  |  |
|  | Male | 1.6 (1.10-2.32) | 0.01* |  |  |  |  |  |
|  | Literate | 2.4 (1.56-3.83) | <0.001* |  |  |  |  |  |
| Knowing that eye tests for spectacles and diabetes are different tests | Literate | 5.0 (3.04-8.31) | 0.002* |  |  |  |  |  |
|  | Being employed | 1.8 (1.2-2.7) | 0.004* |  |  |  |  |  |
