# Supplementary material for: Sociodemographic Factors Influencing Knowledge, Attitudes, Practices, Glycaemic Control and Sight‐Threatening Retinopathy Among Diabetes Patients in Rawalpindi, Pakistan: A Hospital‐Based Cross‐Sectional Study
Source: Public Health Chall. 2026 Sep 23;5(3):e70384. doi: 10.1002/puh2.70384 (PMC13600724; doi:10.1002/puh2.70384)
Supplement: Supplementary file 2 [file PUH2-5-e70384-s002.docx]

**Supplementary file 2.** Results of multiple regression analysis showing significant predictor variables of diabetes-related practice. * represents a significant p-value.

| **Dependent/outcome variable** | **Predictor variable** | | **Odds ratio (95% CI)** | **P-value** |
| --- | --- | --- | --- | --- |
| Number of episodes of uncontrolled diabetes requiring urgent hospital/doctor visit | | Older age | 1.03 (1.00-1.05) | 0.01* |
|  |  | Illiterates | 2.1 (1.40-3.3) | <0.001* |
|  |  | Being unemployed | 1.7 (CI: 1.16-2.56) | 0.007* |
|  |  | Diabetes for up to six years | 2.2 (1.47-3.32) | <0.001* |
| Doing physical exercise | | Younger age | 1.06 (1.04-1.09) | <0.001* |
|  |  | Being on non-insulin treatment | 1.8 (1.22-2.61) | 0.004* |
| Smoking | | Younger age | 1.04 (1.02-1.06) | <0.001* |
|  |  | Males | 2.0 (1.340-2.96) | <0.001 |
|  |  | Diabetes for up to six years | 1.7 (1.13-2.62) | 0.01 |
| Going for diabetes eye check-ups at least once last year | | Younger age | 1.03 (1.01-1.06) | <0.001 |
